# Short- and Long-Term Implications of Human Milk Microbiota on Maternal and Child Health

**DOI:** 10.3390/ijms222111866

**Published:** 2021-11-01

**Authors:** María García-Ricobaraza, José Antonio García-Santos, Mireia Escudero-Marín, Estefanía Diéguez, Tomás Cerdó, Cristina Campoy

**Affiliations:** 1 EURISTIKOS Excellence Centre for Paediatric Research, Biomedical Research Centre, University of Granada, 18016 Granada, Spain; mariaricobaraza@ugr.es (M.G.-R.); joseantonio_gsantos@outlook.es (J.A.G.-S.); m.escuderomarin@gmail.com (M.E.-M.); estefaniadieguezcastillo@gmail.com (E.D.); 2 Department of Paediatrics, School of Medicine, University of Granada, Avda. Investigación 11, 18016 Granada, Spain; 3 Instituto de Investigación Biosanitaria Ibs-GRANADA, Health Sciences Technological Park, 18012 Granada, Spain; 4 Carlos III Health Institute, Avda. Monforte de Lemos 5, 28028 Madrid, Spain; 5 Spanish Network of Biomedical Research in Epidemiology and Public Health (CIBERESP), Granada’s Node, Carlos III Health Institute, Avda. Monforte de Lemos 5, 28028 Madrid, Spain

**Keywords:** human milk, microbiota, health programming, probiotics

## Abstract

Human milk (HM) is considered the most complete food for infants as its nutritional composition is specifically designed to meet infant nutritional requirements during early life. HM also provides numerous biologically active components, such as polyunsaturated fatty acids, milk fat globules, IgA, gangliosides or polyamines, among others; in addition, HM has a “bifidogenic effect”, a prebiotic effect, as a result of the low concentration of proteins and phosphates, as well as the presence of lactoferrin, lactose, nucleotides and oligosaccharides. Recently, has been a growing interest in HM as a potential source of probiotics and commensal bacteria to the infant gut, which might, in turn, influence both the gut colonization and maturation of infant immune system. Our review aims to address practical approaches to the detection of microbial communities in human breast milk samples, delving into their origin, composition and functions. Furthermore, we will summarize the current knowledge of how HM microbiota dysbiosis acts as a short- and long-term predictor of maternal and infant health. Finally, we also provide a critical view of the role of breast milk-related bacteria as a novel probiotic strategy in the prevention and treatment of maternal and offspring diseases.

## 1. Introduction

Human milk (HM) represents the gold standard, providing protective and functional nutrients for the newborn, ensuring healthy growth and development [1,2]. Accordingly, the World Health Organization (WHO) and other international organizations, such as the European Society for Paediatric Gastroenterology, Hepatology and Nutrition (ESPGHAN), recommend that infants should be exclusively breastfed during their first 6 months of life, with breastfeeding continuing until two years of age, combined with complementary feeding [3,4]. However, it is necessary to point out that exclusively breastfed infants present a particular risk of vitamin D deficiency, due to its low concentration in breast milk [5], low maternal vitamin D levels and daily intake, as well as the lack of exposure of newborns and suckling infants to sunlight [6]. Therefore, associations such as the American Academy of Pediatrics and ESPGHAN recommend vitamin D supplementation at doses of 400 IU/day in infants who are exclusively or partially breastfed [7,8].

Unlike its traditional consideration as a sterile fluid, HM is now recognized as an interesting source of potentially probiotic and commensal bacteria such as *Bifidobacterium* spp., *Lactobacillus* spp., *Clostridiales* spp., viral organisms, among others, which may lead to healthy infant gastrointestinal (GI) microbiota and immune system maturation [9,10,11]. Although studies provide strong evidence of the existence of microbes in human milk, their origin and potential role in breastfed infants’ health need to be studied in greater depth, particularly concerning the contribution of HM microbes to the growth and development of infant gut microbiota. In fact, some authors reported that HM microbiota contributes less than previously expected to gut microbiota composition during early life, and virtually nothing at 6 months of age [12,13]. Nevertheless, as human microbiota is essential for optimal host physiology, there is currently great interest in better understanding how HM microbiota dysbiosis could be related to the development of non-communicable diseases in both mother and child [14]. Consequently, methodological advances and more consistent findings are still needed to better understand the uncertain aspects related to HM microbiota origin, composition and function [15], and its potential interaction with other bioactive components. This review aims to summarize and evaluate the current knowledge of HM microbiota, highlighting avenues for future research and potential therapeutic implications for both maternal and child health.

## 2. Human Milk Microbiota: Practical Approaches to Detect Microbial Communities in Human Breast Milk Samples

Culture-dependent approaches were initially used to demonstrate the existence of specific microbiota in HM samples. In this regard, Martin et al. [16] reported the presence of potential probiotic and commensal lactic acid bacteria (LAB), mainly *Lactobacillus gasseri* and *Enterococcus faecium*, in aseptically collected HM samples from healthy women. These bacteria can inhibit the growth of pathogenic bacteria through acetate and lactate production, suggesting that both strains may have a preventive role in neonatal infectious diseases. This experimental methodology has been widely used to identify other bacterial species naturally present in HM, including different LAB strains (*Lactobacillus*, *Lactococcus*, *Leuconostoc*, *Weissella*, etc.), bifidobacteria, facultative anaerobic bacteria, and novel bacterial species as *Streptococcus lactarius* [17,18,19]. However, it fails to detect both strictly anaerobic and non-culturable bacteria, and new experimental approaches have been required to discover and describe the whole HM microbiota.

Thus, the use of culture-independent molecular techniques (qPCR, denaturing/temperature gradient gel electrophoresis, etc.), and Next Generation Sequencing (NGS) technology, have made it possible to overcome these limitations, identifying strictly anaerobic bacteria (*Bifidobacterium* spp., *Bacteriodes* spp., and *Clostridia*) in HM samples from healthy women [18] (Table 1). Hunt et al. [20], using 16S-ribosomal RNA gene pyrosequencing, showed that central node of HM microbiota was mostly formed by *Streptococcus*, *Staphylococcus*, *Serratia* and *Corynebacteria*. Other bacteria, including *Lactobacillus*, *Bifidobacterium*, *Propionibacterium*, *Pseudomonas*, *Bradyrhizobium*, *Sphingomonas* and *Ralstonia*, were also found to a greater or lesser extent in HM samples. Similar results were obtained by Jost et al. [21] using the above-mentioned experimental approach in combination with culture-dependent methods. Interestingly, gut-associated obligate anaerobes bacteria, such as *Bacteroides* and *Clostridia* were also found, which could explain the potential beneficial role of HM and its microbiota on colonic health.

Moreover, culture-independent molecular techniques, such as qPCR plus pyrosequencing and 16S rRNA sequencing, have been crucial for understanding how populations of HM microbiota change over lactation or due to maternal factors during pregnancy or at delivery [22,23]. These findings were further expanded using metagenomic approaches, identifying archaea, viruses, fungi and protozoa as other common members of HM microbial metagenome [24,25]. Although their practical limitations, including the over- or underestimation of certain bacterial groups, dead bacteria detection or targeting the inherent properties of 16S rRNA region, these methodological approaches have allowed the diversity and complexity of HM microbial communities to be evaluated [18]. However, further studies using novel experimental approaches, such as NGS and omics technologies, are still needed, not only to better understand the detailed HM microbiota composition and functions, but also to understand their potential interactions with other milk bioactive compounds.

**Table 1:** Main features of experimental procedures in analysis of bacterial communities.

Method	Description	Advantages	Disadvantages	References
Culture-Dependent Approaches				
Isolation media	Isolation of target bacteria using general or selective culture media; dose–response study of antibiotics effects on bacteria growth	✓ To provide estimates of microbes capable of replicating under experimental conditions. ✓ Low-cost method.	✓ Limited selective media for potential strains of interest. ✓ Inaccurate representation of whole species present in a sample. ✓ Subjective identification. ✓ Need for trained personnel to obtain reliable results.	[26,27]
Culture-Independent Approaches				
Quantitative Polymerase Chain Reaction (q-PCR)	“Classic” target template detection PCR plus fluorescent detection methods to record product formation during each cycle of PCR amplification. Quantification of gene (or transcript) numbers is determined during the exponential phase.	✓ To identify unlimited number of species present in the sample. ✓ To quantify both abundance and expression of taxonomic and functional gene markers. ✓ Safe and fast method.	✓ Artifacts generation by unequal amplification of PCR products (PCR bias). ✓ Unable to identify unknown species.	[28]
DNA microarray	Synthesis of complementary DNA (cDNA) chains from mRNA and subsequent amplification to biotin-labelled complementary RNA (cRNA). Once these fragments are obtained, they will be hybridized with microarray probes and stained for analysis.	✓ Simultaneous detection of thousands of genes or target DNA sequences. ✓ Fast method.	✓ High cost. ✓ Large number of probe designs based on low-specificity sequences. ✓ Lack of control over the pool of analyzed transcripts. ✓ PCR bias.	[29,30]
Flow cytometry	Liquid bacteria suspension is moved into liquid stream (sheath fluid) and then subjected to the effects of a laser, which scatters light in two major directions: Forward angle light scatter (Forward scatter or FSC) or Side-angle light scatter (Side scatter or SSC). Both light signals are converted into electronic signals to analyze bacteria solution based on their fluorescent or light scattering characteristics.	✓ Rapid assay times and data generation. ✓ High numbers of cells analyzed per sample with minimal volume. ✓ High performance.	✓ Low number of fluorescent stains available to analyze bacterial cells’ viability, structure and/or metabolism	[31]
FISH (Fluorescence In Situ Hybridization)	Target DNA is hybridized using specific DNA probe for further fluorescence microscopy analysis	✓ Extensive philogenetic identification. ✓ No PCR bias	✓ Unable to identify unknown species.	[32]
Next-generation DNA sequencing	Identification of nucleotides order in whole genome or targeted DNA/RNA regions using sequencing technology characterized by ultra-high-throughput, scalability and speed.	✓ Detect “unculturable” bacteria. ✓ High precision when exploring the phylogenetic composition of microbial populations. ✓ Detailed analysis of individual genome stretches. ✓ Precise analysis of RNA transcripts for gene expression.	✓ Unable to analyze metabolic activity and bacterial viability. ✓ Measurement of the relative abundance of bacterial populations rather than absolute abundance. ✓ High cost ✓ Need for qualified personnel and massive amount of work to analyze the obtained results.	[31,33]

## 3. Composition and Potential Origin of the HM Microbiota

Different studies have reported the existence of “core” HM microbiota, mostly formed by *Staphylococcus*, *Streptococcus* and *Propioninacterium* [21,34,35,36], although its composition can vary between 2 and 18 taxa, including other members at lower abundances such as *Bifidobacterium*, *Veillonella*, *Rothia*, *Lactobacillus*, *Corynebacterium*, *Ralstonia*, *Acinetobacter*, *Acidovorax*, *Pseudomonas*, *Bacteroides*, *Clostridium*, *Escherichia/Shigella*, *Gemella*, and *Enterococcus* [37]. This lack of consensus in HM microbiota composition seems to be due to differences in both methodologies (sample collection and stored, DNA extraction protocols, 16S rRNA sequenced region) [38] and study populations (geographical and lifestyle factors) [39]. However, it seems clear that “core” HM microbiota acquisition occurs gradually over lactation, and this could potentially drive healthy gastrointestinal microbiota development and subsequent optimal later-life health [37,40].

There are different questions about the origin of HM microbiota. Scientific evidence supports a pre-existing mammary gland microbiota formed before pregnancy and lactation, thus acting as a basis for HM microbiota establishment [41,42,43]. Once set, the existence of multiple microbial sources, such as maternal skin and GI tract, as well as infant oral cavity, could direct the development of HM microbiota. This fact would not only explain the differences in composition between mammary gland and HM microbiota, but also its dynamic composition during breastfeeding [37]. Nevertheless, to date, this is still under discussion; in fact, some studies have detected human skin bacteria, mainly genus *Staphylococcus*, *Propionibacterium* and *Corynebacterium*, in HM samples, suggesting the ability of maternal skin microbiota to colonize the mammary gland [20]. However, other findings fail to identify this microbial source. Thus, although LAB were found in both HM and mammary areola, these bacteria showed different DNA profiles according to the analyzed source [16]. Similarly, there were also significant differences in *Streptococcus* and *Propionibacteria* abundance between microbial communities present in the HM and maternal skin [21]. It is also important to highlight that the bacterial genera shared between HM and maternal skin also seem to be related to GI tract and mouth [34], thus reinforcing the need for further studies to clarify the real contribution of maternal skin to HM microbiota. Interestingly, HM microbiota origin could be also explained by bacterial retrograde flow between breast milk and infant oral cavity. This assumption is not only suggested by the presence of typical oral bacteria species, mainly *Streptococcus*, *Rothia* and *Gemella*, in HM samples [44], but also by the significant differences in oral microbiota composition found between formula-fed and breastfed infants [45].

Finally, there is growing interest in entero-mammary pathway to explain the potential HM microbiota origin, which is founded by the presence of typical GI bacteria (*Bifidobacterium*, *Veillonella*, *Bacteroides*, and *Clostridium*, among others) in breast milk [12,21,34,46,47]. This hypothesis is also supported by studies performed in mice models, which suggest that dendritic cells (DC) can regularly reach the intestinal epithelium and selectively recognize commensal gut bacteria to move them to the mammary gland through lymphatic system [36]. Zhou et al. [48] also found that DC shared bacterial signatures with those reported in the intestine, lymph nodes and breast milk in lactating mice. Furthermore, interventional studies performed in lactating women have reported that HM microbiota composition can be selectively modified by diet or pro- and prebiotic treatment, emphasizing the potential maternal GI origin of HM microbiota [49,50]. Recent studies also suggest that the entero-mammary pathway is involved in immune cell circulation, which may partially explain the immunomodulatory role of HM, which is discussed in more detail below. In this sense, Ikebuchi et al. observed a higher proportion of CD4+ and CD8+ T cells in milk compared to mammary gland in mouse model [51]. Moreover, CD8+ T cells showed increased expression of claudin polymerization-associated genes (*cldn3* and *cldn7*), as well as the *tjp1* gene, which is related to the biosynthesis of tight junction protein ZO-1. This expression profile, observed in CD8+ T cells (and probably in CD4+ T, γδT, and NK cells), allows immune cells to selectively translocate through the tight junction region to milk. Consequently, when the infant is breastfed, T cell populations could migrate to lymphoid tissues, thus reinforcing neonatal-cell-mediated immunity [52]. Therefore, CD4+ T-cell-mediated adaptive immune response is crucial to maintain intestinal immune homeostasis against food molecules and non-harmful microbial components [53], while CD8+ T-cell-mediated response plays a key role in protection against intracellular pathogens, thereby maintaining an optimal balance in the gut microbial community [54].

Taken together, further studies are not only needed to better evaluate the potential contributions of different sources to HM microbiota composition, but also to clarify whether the bacterial communities present in mammary gland are a true permanent microbiota or constantly provided by external sources. It will be also of interest to explore the probable interactions between HM microbiota and immune components present in HM.

## 4. Potential Factors That Influence HM Microbiota Composition

**Figure 1:**
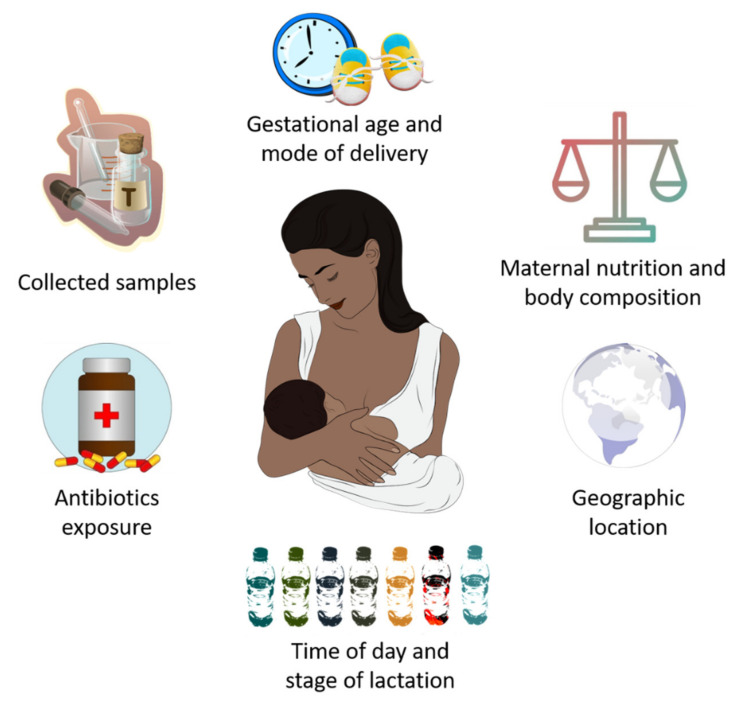
Potential factors that influence breast milk microbiota composition.

In recent years, it has been established that HM microbiota composition is highly influenced by genetic, maternal and early-life factors (gestational age, delivery mode, maternal nutrition and body composition, time of day and stage of lactation, intake of antibiotics, and geographic location), which cause substantial inter-individual variation in its composition [55] (Figure 1).

In this line, both gestational age and mode of delivery can modify the abundance of certain bacteria in HM microbial community. Khodayar-Pardo et al. [56] observed a lower abundance of *Lactobacillus* spp. (*L. fermentum* and *L. salivarius*) and *Bifidobacterium* spp. in HM samples from women who had a cesarean delivery with respect to those who gave birth vaginally. Moreover, human milk *Bifidobacterium* concentrations were increased in mothers who had a term delivery compared to those who had a preterm birth. On the other hand, Cortés-Macías et al. [57] reported that C-section delivery and antibiotic exposure caused compositional changes in the HM microbial community, in terms of lower abundances of *Lactobacillus*, *Bacteroides*, and *Sediminibacterium* genera.

Regarding the influence of maternal diet and body composition on HM microbiota composition, the results achieved to date are limited and indicate a need for further research. Padilha et al. [58] observed that HM microbiota diversity was associated with vitamin C intake during pregnancy, while human milk *Bifidobacterium* concentration was positively correlated to polyunsaturated and linoleic fatty acids intake during lactation.

Interestingly, maternal BMI seems to have an impact, either positive or negative, on the diversity of certain bacteria phylum, but not on overall α-diversity [59]. However, the potential influence of maternal diet and body composition on HM microbiota composition is still controversial. In this line, studies conducted to date suggest that both the aforementioned factors seem to be associated with changes in the HM profiles of oligosaccharides (HMOs), fatty acids, proteins, hormones, immune cells and antibodies [60,61,62]. These changes could alter the milk microenvironment and, ultimately, microbial community composition [59].

Recently, scientific research has focused on the role of circadian rhythms in HM microbiota composition. Corona-Cervantes et al. [63] showed changes in Shannon diversity index during the day, as well as a predominance of *Proteobacteria*, *Actinobacteria* and *Firmicutes* at night, in HM samples from healthy women who had vaginal deliveries. Despite the limitations of this study, the obtained findings seem to suggest that HM microbiota could follow a circadian rhythm, with dynamic changes in its composition depending on the time of day.

Lactation stages have been also identified as a novel factor that can contribute to the remodeling of HM microbiota composition. Gonzalez et al. [64] found that HM samples collected during early lactation (6–46 days post-partum) were rich in *Staphylococcus* and *Streptococcus* spp., both related to infant oral and intestinal tract, while aromatic compound degradation-related species such as *Sphingobium* and *Pseudomonas* were prevalent in HM samples collected at the late stage (109–184 days post-partum). Likewise, Moossavi et al. [59] showed that HM microbiota composition, but not α-diversity, was related to lactation stage within the period of study (17 ± 5 weeks). Moreover, they also showed that indirect breastfeeding decreased the relative abundance of *Bifidobacterium* but increased relative abundance of potential pathogens, including *Enterobacteriaceae* and *Pseudomonas*. Similar to other studies [21,65], these authors also suggest that the collection method is an extrinsic factor influencing HM composition analysis.

It is known that geographic location influences HM composition, including levels of micro- and macronutrients, bioactive compounds, and immunological factors, and thus ultimately also affects HM microbiota composition [66,67]. In fact, after analyzing HM samples belonging to 117 Chinese mothers from three different geographic locations, Wan et al. [68] reported that microbial diversity and richness during lactation were potentially related to maternal geographic location.

## 5. Human Milk Microbioma Functions and Activity

The recent technological advances discussed above have allowed the potential functions of human-milk-associated bacteria in promoting host health to be identified. In this line, the results obtained from diverse studies suggest that HM microbiota may influence infant gut microbial colonization through vertical mother-child bacterial transmission [34,69,70,71]. In support of this hypothesis, both HM and breastfed infants’ stool samples seem to share specific bacteria patterns (mainly *Lactobacillus* spp., *Lactobacillus plantarum*, *Bifidobacterium breve* and *Bifidobacterium longum* subsp. *longum*), with significant differences also seen compared to strains obtained in faeces from formula-fed infants [34,69,70,71,72]. Interestingly, this similarity in microbial composition remains throughout the first year of life [73]. The vertical transmission of HM microbes to infant gut would not only be facilitated by milk’s own characteristics, but also by specific microbial features that could allow them to survive the GI environment. On the one hand, the neutral pH of breast milk counteracts the acidity of the GI tract during lactation. Moreover, HM is also rich in non-digestible carbohydrates (HMOs) that facilitate commensal bacterial growth [74]. On the other hand, members of the *Bifidobacteria* genus present in HM might colonize and persist in the GI tract due to the specific structural features present in its surface combined, with crucial molecular tools specifically designed to respond to environmental changes [75,76]. Among them, *Bifidobacteria* has the ability to resist the acidic environment of GI tract through its F0F1-ATPase activity, which is responsible for active proton extrusion and the maintenance of pH homeostasis [77]. Nevertheless, despite this evidence, the potential influence of HM bacteria on infant gut colonization is still under review as studies carried out to date show inconsistent findings. In fact, Pannaraj et al. [12] reported that the contribution of HM to infant gut microbiota in breastfed infants ranged between 15 and 20%, while Williams et al. [13] found that HM bacteria represented only about 5% to the infant gut microbiome on day 2 of life, but virtually nothing at 6 months of age. Consequently, further studies are still needed to better understand the potential role of HM bacteria on infant gut colonization.

It is also important to highlight that HM microbiota might be involved in the microbiota establishment in other niches. For instance, HM microbiota might drive oral microbiota development, as suggested by the higher abundances of HM-related bacteria in oral samples from breastfed infants compared to those who received infant formula [13,78,79]. Likewise, HM microbiota seems to influence upper respiratory tract (URT) microbiota establishment during early life, thus supporting the protective effects of HM against infants URT infections. In fact, URT microbiota from breastfed infants is mainly formed by LAB, but there is a low abundance of *Staphylococcus* and anaerobic bacteria, with the latter being predominant in URT microbiota from formula-fed infants [80,81].

Due to its hypothetical role in GI microbiota establishment, HM microbial may have other potential functions in the infant gut. Studies suggest that HM-related bacteria protect against gastrointestinal infections through different mechanisms including: (i) growth inhibition of pathogenic bacteria by competitive exclusion; (ii) production of antimicrobial compounds such as hydrogen peroxide (H_2_O_2_); (iii) enhancing intestinal barrier protection via increased mucine production, lower intestinal permeability, and upregulation of detoxifiying enzymes [82]. Consequently, HM-related bacteria strains belonging to Lactobacillus (*L. salivarius* CECT5713 and *L. fermentum* CECT5716) and *Staphylococcus epidermidis*, have emerged as promising therapeutic agents in the treatment of child gastrointestinal infections [82,83,84].

Scientific evidence also suggests a potential metabolic role of HM microbiota in infants; as an example, both lactobacilli and bifidobacteria have the ability to break down non-digestible HMOs into butyrate, which is not only used for colonocytes as energy source, but also has modulatory effects on intestinal health [85,86]. Additionally, HM microbiota seems to be involved in nutrient metabolism and synthesis, as suggested by the fact that stool samples from breastfed infants showed a significant increase in carbohydrates, amino acids and nitrogen metabolism, as well as cobalamin synthesis, compared to those obtained from formula-fed infants [87].

Certain bacteria strains provided by breast milk, along with other HM bioactive compounds (nutritional components, hormones, growth factors, neuropeptides, cytokines and nucleotides) can maturate and modulate immune responses in neonates and infants, thus protecting them against asthma, allergies and other non-communicable diseases [88]. In vitro models suggest that *Lactobacillus salivarius* CECT5713 and *Lactobacillus fermentum* CECT5716 could have potent immunomodulatory effects by regulating the activation of NK cells, CD4+/CD8+ T cells and regulatory T cells [89]. Moreover, compared to formula-fed infants, the immune response observed in breastfed infants is largely based on regulatory T cells and TH1/TH2 balanced responses [90]. Other human-milk-related bacteria strains, including *Lactobacillus gasseri* CECT5714 and viridans streptococci, also seem to have a protective role in allergic conditions, mainly cow´s milk protein allergy/intolerance [91] and atopic eczema [92], respectively. Overall, these studies suggest the possible immunomodulatory role of HM microbiota in infants.

## 6. Short- and Long-Term Implications of HM Microbiota on Maternal and Child Health

Regardless of its origin, the existence of commensal microbes in HM might have a beneficial role in the health of mothers and their newborn infants. In fact, research carried to date points to a potential influence of HM microbiota on health outcomes [93,94]. However, further studies are still needed to obtain stronger scientific evidence, particularly considering that HM not only contains commensal bacteria, but also many other immune, nutritional and bioactive factors that may influence maternal and child health.

### 6.1. Maternal Pathologies and Human Milk Microbiota Dysbiosis

It is established that breastfeeding provides short- and long-term positive effects on maternal health, including better postpartum recovery, lower risk of breast and ovarian cancer, and a reduced incidence of cardiovascular and autoimmune diseases [95,96]. Interestingly, clinical and scientific evidence also suggests a bidirectional interaction through which maternal health can modify HM microbiota composition.

Mastitis is a common inflammatory condition that affects 33% of lactating women and causes pain during lactation, redness of the breast and fever, ultimately leading to decreased milk production and subsequent early suppression of breastfeeding [97]. There is evidence suggesting an association between this inflammatory condition and HM microbiota dysbiosis in terms of low microbial diversity, decreased relative abundances of commensal bacteria (*Lactococcus* and *Lactobacillus*) and the establishment of opportunistic pathogens such as *Staphylococcus*, *Streptococcus* and *Corynebacterium* [98,99]. In fact, acute mastitis is widely caused by *S. aureus*, reaching concentrations of 4.0 log_10_ colony-forming cells (cfu)/mL in milk of acute mastitis-suffering women compared to concentrations from 1.5 to 3 log_10_ cfu/mL reported in the milk of healthy women. However, other potentially pathogenic strains, including coagulase-negative *Staphylococci*, *S. epidermidis* and *Corynebacterium*, also lead to subacute, chronic or granulomatous mastitis in lactating women, respectively [100,101]. As these bacteria are often resistant to antibiotic therapy, promising strategies for mastitis treatment are currently based on the use of *Lactobacillus* strains isolated from the human milk of healthy women. Clinical trials published to date indicate that oral intake of different *Lactobacillus* strains isolated from human milk, either alone, such as *L. fermentum* CECT5716 and *L. salivarius* CECT5713 [102], or combined (*L. salivarius* CECT5713 plus *L. gasseri* CECT5714) [49], reduces the counts of pathogenic bacteria and improves mastitis symptoms after 14–21 days of treatment, thereby emerging as promising treatment for lactational infectious mastitis when antibiotic treatment fails. Interestingly, both *L. fermentum* CECT5716 and *L. salivarius* CECT5713 were also found in HM samples of treated women, suggesting that both strains are able to recolonize the mammary gland to reduce and reverse mastitis-associated dysbiosis [102]. Further studies have been performed to better understand the key biomarkers and potential mechanisms involved in this probiotic effect. Overall, these studies showed that *Lactobacillus*-based probiotic treatment did not affect milk macronutrient composition, but was associated with specific microbiological, immunological and metabolomic markers related to the improved integrity of mammary gland epithelia [103,104]. Moreover, a recent study also suggest that probiotic treatment might act on specific genes involved in inflammatory and cell-growth signaling pathways, thus opening new avenues for research based on specific responsive molecular targets [105]. Finally, daily oral intake of HM-related *L. salivarius* PS2 between week 30 of gestation and delivery, significantly reduced the incidence of mastitis in women who suffered this pathology in previous pregnancies, compared to those who received a placebo during the same period [98].

The potential protective role of HM microbiota in breast health is also indirectly suggested by the close link between the microbial communities present in mammary tissues and risk of breast cancer. In this line, Urbaniak et al. [106] reported cancer-related dysbiosis characterized by a significantly lower abundance of LAB, but increased abundance of *Bacillus* spp., *Staphylococcus epidermidis*, family *Comamonadaceae* and Enterobacteria such as *Escherichia coli*. However, the potential mechanisms through which microbiota dysbiosis could contribute to breast cancer are still unknown. On the one hand, this effect could be explained due to the ability of *S. epidermidis* and *E. coli* to induce DNA damage by double-strand breaks [106]. Moreover, breast-cancer-associated microbial dysbiosis could downregulate the host immune system, which, in turn, leads to a permissive environment for breast tumorigenesis [107]. Xuan et al. [108] found a lower abundance of *Sphingomonas yanoikuyae* in breast tumor tissue, a gram-negative bacteria involved in immune cell activation and the inhibition of tumor growth. In a similar study, nipple aspirate fluid from breast cancer women was rich in genus *Alistipes* and other bacteria with β-glucuronidase enzymatic activity, which is associated with profound changes in estrogen metabolism and an increased risk of breast cancer [41]. It is also important to note that the use of chemotherapy to treat breast cancer might alter the bacterial communities present in HM and mammary tissues, reducing these potentially beneficial bacteria for mother and infant health [43]. Despite this evidence, further studies are still needed to clarify whether these bacteria strains could grow into a tumorigenic environment or whether they are a direct cause of breast cancer.

Other maternal pathologies also seem to be accompanied by HM microbiota dysbiosis. For instance, González et al. [109] showed that breast milk of human immunodeficiency virus (HIV)-infected women presented increased bacterial diversity and *Lactobacilllus* spp. frequency, but its content in *S. hominis* and *S. aureus* was lower compared to breast milk of healthy women. Nevertheless, no evidence for HIV-related microbial dysbiosis was found in other studies [110]. These contradictory findings may be explained by methodological and population differences between studies, making it difficult to identify whether changes in HM microbiota composition were a response to maternal disease or vice versa. Similar conclusions have been obtained in studies that analyzed HM microbiota composition in women who suffer from allergies or celiac disease. In both cases, lower relative levels of *Bifidobacterium* and *Bacteroides* were found in breast milk samples, but there were significant differences in dietary habits between healthy and unhealthy women [111,112].

Special mention should be made to the potential relationship between HM and coronavirus disease 2019 (COVID-19). Although HM microbiota dysbiosis has not yet been found in women positive for SARS-CoV-2, recent evidence suggest that GI microbiota is profoundly altered in COVID-19 patients, particularly in terms of reduced bacterial diversity and lower levels of commensal bacteria with immunomodulatory role (*Faecalibacterium prausnitzii*, *Eubacterium rectale* and *Bifidobacterium*), as well as increased growth of potentially pathogenic *Enterococcus* strains [113,114]. These changes in GI microbiota composition seem to be positively related to cytokine storm intensity and subsequent disease severity [114], and preliminary results suggest that therapeutic strategies focused on GI microbiota modulation using pro- and synbiotics (mainly *Lactobacillus* spp. and *Bifidobacterium* spp.) could be effective in the prevention and treatment of severe COVID-19 [115]. Interestingly, noting the entero-mammary origin, it is plausible that COVID-19 disease also involves dynamic changes in HM microbiota composition. Moreover, due to the hypothetical role of HM microbiota in the establishment of infant GI microbiota, breastfeeding could have a potential protective effect on severe COVID-19-related dysbiosis in infants. While both assumptions require further research, there are different approaches, emphasizing that mothers infected by SARS-CoV-2 can breastfeed their infants, with the necessary precautions, in order to transmit HM’s protective properties against COVID-19 disease [116,117,118,119]. In fact, unlike other viruses- such as HIV and human cytomegalovirus, which can be transmitted to infants via breast milk, Bäuer et al. reported that HM samples obtained from mothers with SARS-CoV-2 infection and/or those who have recovered from COVID-19, showed no presence of SARS-CoV-2 RNA [120]. Interestingly, these authors also observed that HM could provide passive immunity to breastfed infants via the transfer of SARS-CoV-2 spike-protein-specific antibodies. Similarly, Demers-Mathieu et al. found a positive correlation between antigens and secretory antibodies in breast milk samples from mothers with confirmed COVID-19 PCR, characterized by higher levels of S2 subunit SARS-CoV-2-specific IgG, while SIgA and SIgM were polyreactive and cross-reactive to S1 or S2 subunit SARS-CoV-2 [121]. In conclusion, the data discussed here seem not only to support the breastfeeding recommendations during the COVID-19 pandemic, but also its potential beneficial role for mothers and their offspring in the prevention of severe COVID-19 disease [122]. However, as mentioned above, further studies are required to better understand the role of both HM and GI microbiota in the physiopathology and management of COVID-19.

Lastly, there is growing interest in evaluating the effects of maternal metabolic conditions during pregnancy on the composition and activity of HM microbiota, as well as its potential associations with later maternal and child health status. In this regard, it is now established that maternal obesity and gestational diabetes mellitus (GDM) involve gut microbiota dysbiosis which, if we consider the entero-mammary pathway as a potential origin of HM microbiota, might result in HM microbiota dysbiosis. Thus, the gut microbiota of obese women is characterized by their lower diversity and higher *Firmicutes*:*Bacteroidetes* ratio with respect to lean women [123]. Similar changes were also found in gut microbiota composition in women affected with GDM, including lower α-diversity, changes in β-diversity, higher *Firmicutes*:*Bacteriodetes* ratio, increased prevalence of gram-negative bacteria, and reduced levels of potential probiotic bacteria [124]. Therefore, these dynamic changes might not only alter HM microbiota composition, but also generate an “obesogenic” environment in infant gut, thus increasing infant obesity risk [123,124]. However, there is limited knowledge about the possible effects of maternal metabolic conditions on HM microbiota composition. Studies conducted to date seem to indicate that both maternal pre-pregnancy obesity and excessive gestational weight gain were related to lower diversity and *Bifidobacterium* abundance, but increased counts of *Lactobacillus* and *Staphylococcus* in milk samples [22,125]. These characteristics in HM microbiota composition were also related to changes in immunological biomarkers [125], which may further explain the plausible link between higher risk of child and maternal obesity and HM microbiota dysbiosis. Recent studies have focused on analyzing the combined impact of both maternal metabolic conditions on HM composition. LeMay-Nedjelski et al. [126] found that milk samples obtained from obese mothers with GDM or impaired glucose tolerance contained higher levels of *Gemella*, compared to normal-weight mothers. Moreover, the colostrum samples of obese mothers with GDM showed higher microbial diversity and increased levels of amino acid and carbohydrate metabolism-related bacteria [127]. However, the HM microbiota composition reported in these studies was also affected by other confounders, including type of delivery, use of antibiotics, ethnicity and infant sex [126,127]. Consequently, further studies are required to better evaluate potential mechanisms by which HM microbiota from mothers suffering obesity and GDM may influence later health and development.

### 6.2. Role of Human Milk Microbiota on Child Health

Several clinical trials have described the potential benefits of HM in infants who suffer from necrotizing enterocolitis (NEC), gastrointestinal disorders, celiac disease, obesity, dermatitis, asthma, and infection-related processes such as surgical procedures and chemotherapy [128,129]. These health effects are largely due to HM’s composition, which is rich in nutritional, immune and bioactive compounds. Furthermore, the presence of commensal and potentially probiotics bacteria could also be an important factor in explaining the protective effects of HM on infant health. For instance, some authors suggest that *Bifidobacterium breve*, a common member of the microbiota of breastfed infants, could be key to promoting healthy GI microbial colonization due to its ability to use HMOs, thus possibly protecting against infection and modulating immune system maturation [130,131]. However, it is important to point out that further studies are still needed to accurately understand the potential implication of HM microbiota on infant health, as well as the possible biological mechanisms and interactions with other bioactive compounds through which HM microbiota could exert these potential protective effects on child health.

NEC is a major cause of acquired intestinal morbidity and neonatal death, especially in preterm infants [132]. Although a clear dysbiosis pattern has not yet been reported, the obtained findings suggest that preterm infants suffering from NEC or nosocomial sepsis showed dynamic changes in gut microbiota composition (mainly decreased bacteria diversity and high levels of potentially pathogenic bacteria such as *Proteobacteria* and *Clostridium perfringes*), compared to healthy infants [133,134]. Due to its possible ability to modulate infant gut microbiota, human milk feeding has emerged as promising strategy to reduce the risk of NEC [135]. In addition to its high nutritional, the preventive role of HM could be explained by its high content of commensal beneficial bacteria, including *Bifidobacteria*, *Lactobacillus*, and *Streptococcus*. Interestingly, these bacteria strains showed both species-specific probiotics effects and wider preventive effects when combined [135,136]. Breastfeeding should be also encouraged in preterm infants due to its high HMO concentration, which favors commensal bacteria growth in the gastrointestinal tract. This would explain why breastfed infants respond better to probiotic treatment than formula-fed infants [127]. For mothers unable to produce sufficient breast milk to meet the nutritional needs of their premature infants, pasteurized donor milk supplemented with the mother´s own milk is highly recommended to support optimal gut microbiota maturation, thus improving premature infant health [137]. In combination with this modulatory role in infant GI microbiota, HM can also decrease the risk of neonatal NEC through anti-inflammatory mechanisms related to the inhibition of the NF-κB signaling pathway. In fact, HM reduces IL-1β-induced activation of the IL-8 gene, an NF- κB-dependent, pro-inflammatory cytokine that is crucial for NEC pathophysiology. This anti-inflammatory effect seems to be related not only to increased IκBα synthesis, a key inhibitor of the NF-κB pathway, but also to a decrease in its 26S proteosome-dependent degradation [138]. Taken together, these results suggest that breastfeeding, due to its complete nutritional composition, should be taken into account as a protective and therapeutic strategy to reduce the risk of NEC and other inflammatory bowel diseases.

Human-milk-related beneficial bacteria also seem to have a protective effect on minor gastrointestinal disorders in healthy infants; as an example, the intake of infant formula enriched with *L. fermentum* CECT5716 Lc40 or *B. breve* CECT7263, both previously identified in breast milk, reduced both the frequency and recovery time of GI infection-associated diarrhea and infant colic-associated crying, respectively [139]. However, other HM compounds such as HMOs are also implicated in the prevention of infant gastrointestinal disorder. Thus, 2′-fucosyllactose (2′-FL), a HMOs related to Secretor gene *fut2* [140], plays a protective role in diarrhea caused by *Campilobacter jejuni* [141]. Similarly, fucosyltransferase enzyme (FUT3), associated with the Lewis-Secretor gene [140], is involved in the synthesis of different types of HMOs with potent in vitro antimicrobial activity against Group B Streptococcus (GBS), potentially reducing the risk of neonatal infection [142].

The protective role of breastfeeding on the incidence and severity of infant atopic disorders (AD) and asthma has gained a lot of research interest as the prevalence of both pathologies is increasing globally [143,144]. However, the results obtained to date are controversial. In this respect, Orivuori et al. reported that soluble IgA (sIgA) levels in breast milk were associated with microbial-load-related environmental factors but not with breastfeeding duration. Interestingly, sIgA levels during the first year of life were related to lower risk of AD up to between 2 and 4 years, but associations between sIgA levels and risk of AD or asthma were not found at 6 years [145]. On the other hand, the results obtained from exhaustive review and meta-analysis showed that children who were breastfed longer had a lower risk of developing asthma and eczema up to 2 years of age, although this risk increased with infant’s age [146]. Conversely, Kong et al., using non-targeted metabolic analysis in mouse model, identified the long-chain saturated fatty acids (LCSFA) present in breast milk as damage-associated molecular patterns (DAMPs); thus, breast milk intake was related to increased levels of inflammatory Group 3 innate lymphoid cells (ILC3) in gut, with increases in the production of IL-17 and IL-22, which may migrate to the skin and increase the risk of AD [147].

Based on its immunomodulatory role, human-milk-related LAB could have a promising therapeutic effect on infant allergic conditions [148]. According to this assumption, in vitro studies suggest that *L. salivarius* CECT5713 and *L. fermentum* CECT5716 isolated from breast milk are potent activators of NK cells, but their effects seem to be moderate on CD4+, CD8+ and regulatory T cells, and seriously limited on T cells activation. Moreover, both potentially probiotic strains could modulate the cytokine patterns, favoring Th1 immunity response and enhancing both innate and acquired immune responses [89]. Interestingly, the plausible protective role of HM in allergic conditions could be related to the low contents of *Bifidobacteria* found in breast milk from allergic women [149]. Thus, maternal probiotic treatment with *Lactobacillus* spp. and/or *Bifidobacterium* spp. aiming to modulate HM microbiota should be considered a useful tool for the prevention or treatment of allergic conditions, although questions about species-specific and dose-dependent effects, time of administration and treatment duration remain unsolved [149].

Finally, Gough et al. [150] found a lower bacterial diversity and high concentrations of *Acidaminococcus* genus in gut microbiota from infants who suffer from severe linear growth retardation. Considering its potential role in healthy infant gut colonization, these findings might suggest that the beneficial features of HM microbiota could determine optimal infant growth and development, although direct evidence has been not reported to date.

Overall, the findings discussed here suggest that the complex modulation of infant gut microbiota through breast milk could have beneficial effects on infant health. These benefits are especially important in preterm infants since their GI microbiota are rich in potentially pathogenic bacteria. However, the available evidence is sparse, and further studies should be carried out to better understand the role of HM microbiota in infant health, which would allow us to identify novel HM-related beneficial strains as promising therapeutic tools for the treatment of microbiota-dysbiosis-related disorders.

## 7. Conclusions

1. Advanced experimental approaches have made possible to identify the existence of an HM microbiota “core” primarily consisting of *Lactobacillus*, *Staphylococcus*, *Propionibacterium* and *Streptococcus*. An HM bacterial “core” is gradually acquired over lactation, although several maternal factors contribute to its composition, including gestational age, delivery mode, maternal nutrition and body composition, antibiotics intake and geographic location.
2. There is growing evidence supporting the mother-to-infant vertical transmission of HM-related bacteria. However, the original source of such bacteria is still unclear, with maternal skin and GI tract, as well as infant oral cavity, serving as potential HM microbiota sources.
3. Independently of its origin, HM microbiota may promote optimal health status in mothers and their infants via the broad array of potential functions related to the healthy establishment of gut microbiota, growth inhibition of pathogenic bacteria, and its modulatory effects on both metabolic pathways and immune responses.
4. HM microbiota dysbiosis seems to be associated with specific disease conditions, both maternal (mastitis, breast cancer, and metabolic diseases during pregnancy) and in infants during their early life (necrotizing enterocolitis, allergies, infections, alterations in growth and development, among others).
5. Finally, although further studies are needed to better understand the protective role of HM microbiota, the isolation of different beneficial strains present in breast milk, mainly belonging to *Bifidobacterium*, *Lactobacillus*, and *Streptococcus*, could provide alternate therapeutic options against those disorders related to HM microbiota dysbiosis in which antibiotic-based therapy does not produce the desired effect.

## Data Availability

Not applicable.
